# Transparent Low Electrostatic Charge Films Based on Carbon Nanotubes and Polypropylene. Homopolymer Cast Films

**DOI:** 10.3390/polym10010055

**Published:** 2018-01-09

**Authors:** Zoe Vineth Quiñones-Jurado, Miguel Ángel Waldo-Mendoza, José Manuel Mata-Padilla, Pablo González-Morones, Juan Guillermo Martínez-Colunga, Florentino Soriano-Corral, Víctor Javier Cruz-Delgado, Ronald Francis Ziolo, Carlos Alberto Avila-Orta

**Affiliations:** 1 Innovación y Desarrollo en Materiales Avanzados A.C., Grupo POLYnnova, Carr. San Luis Potosí-Guadalajara 1510, Nivel 3, Local 12, Lomas del Tecnológico, San Luis Potosí S.L.P. C.P. 78211, Mexico; zoe.vineth@polynnova.mx (Z.V.Q.-J.); miguel.waldo@polynnova.mx (M.Á.W.-M.); 2 CONACyT—Centro de Investigación en Química Aplicada, Blvd. Enrique Reyna H. 140, Col. San José de los Cerritos, Saltillo, Coahuila C.P. 25294, Mexico; jose.mata@ciqa.edu.mx; 3 Centro de Investigación en Química Aplicada, Blvd. Ing. Enrique Reyna H. 140, Col. San José de los Cerritos, Saltillo, Coahuila C.P. 25294, Mexico; pablo.gonzalez@ciqa.edu.mx (P.G.-M.); guillermo.martinez@ciqa.edu.mx (J.G.M.-C.); florentino.soriano@ciqa.edu.mx (F.S.-C.); rziolo@cs.com (R.F.Z.)

**Keywords:** polymer films, extrusion, cast, carbon nanotube, antistatic, optical properties

## Abstract

Use of multi-wall carbon nanotubes (MWCNTs) in external layers (A-layers) of ABA-trilayer polypropylene films was investigated, with the purpose of determining intrinsic and extrinsic factors that could lead to antistatic behavior of transparent films. The incorporation of 0.01, 0.1, and 1 wt % of MWCTNs in the A-layers was done by dilution through the masterbatch method. Masterbatches were fabricated using isotactic polypropylene (iPP) with different melt flow indexes 2.5, 34, and 1200 g/10 min, and using different ultrasound assist methods. It was found that films containing MWCNTs show surface electrical resistivity of 10^12^ and 10^16^ Ω/sq, regardless of the iPP melt flow index (MFI) and masterbatch fabrication method. However, electrostatic charge was found to depend upon the iPP MFI, the ultrasound assist method and MWCNT concentration. A percolation electron transport mechanism was determined most likely responsible for this behavior. Optical properties for films containing MWCNTs do not show significant differences compared to the reference film at MWCNT concentrations below 0.1 wt %. However, an enhancement in brightness was observed, and it was attributed to ordered iPP molecules wrapping the MWCNTs. Bright transparent films with low electrostatic charge were obtained even for MWCNTs concentrations as low as 0.01 wt %.

## 1. Introduction

Polymer-based packaging films are widely used in the food industry to preserve all kinds of foods, such as cookies, bread, meat, beverage liquids, etc. One of the polymers used in an extensive manner in the fabrication of packaging films is isotactic polypropylene (iPP) and its copolymers with polyethylene, due to its low cost, good mechanical properties and excellent optical properties [1]. However, these types of polyolefins possess a non-polar structure showing hydrophobic behavior. It is well known that hydrophobic polymers generate static electricity during processing, causing dangerous explosions and attract dust, giving an old-like appearance to the food package [2,3]. Nowadays, the static electricity problem in iPP films is usually solved by adding antistatic additives in a polymer carrier (masterbatch), such as with additives having molecules with one hydrophilic end that migrates to the surface of the film, due to its repellence with hydrophobic iPP [3]. Once on the film surface, the hydrophilic end attracts water molecules forming a low resistance antistatic layer (10^10^–10^12^ Ω/sq). However, due to their migrating nature, additives are totally expelled from the film, thus, the antistatic effect is hindered or abates over the time. Therefore, there is an industrial need for the development of new non-migrating antistatic additives in polymer carriers that are capable of maintaining the antistatic effect over longer periods of time [4].

Carbon nanoparticles (CNPs), such as carbon black (CB), carbon nanotubes (CNTs), carbon nanofibers (CNFs), and graphene (G), among others, are good candidates for this purpose since they are excellent charge carriers [5,6,7]. When introduced into iPP or other thermoplastic polymers, a percolation curve is typically observed [8,9]. However, depending on the intrinsic properties of iPP and the corresponding CNP, as well as on the processing conditions [10,11], percolation (conductive pathway) can be obtained at low or high loadings of CNPs where dispersion plays a key role, i.e., a given polymer/CNP nanocomposite can transport electrical charge, however, depending on the loading, it can look greyish or black [12,13]. Therefore, the challenge is to achieve a flexible antistatic film using CNPs that is transparent. For this purpose, a minimum amount of CNPs must be used in order to have a minimum effect on the optical properties of the films. In this sense, it is the aim of this study to determine the feasibility of using masterbatches based on iPP/multi-wall carbon nanotube (MWCNT) prepared with different ultrasound-assist methods, and with iPPs with different melt flow index (MFI) [14,15], in the fabrication of cast films with a minimum effect on the optical properties. For this purpose, electrical and optical properties of the materials were determined. Although it is well known that in many industrial applications, PP films are bioriented (BOPP), the scope of this study is limited to cast films.

## 2. Materials and Methods

### 2.1. Materials

MWCNT masterbatches (10 wt %) used in this work were fabricated at CIQA (Saltillo, Mexico). The masterbatches were prepared using different iPPs as shown in Table 1, while the base polymer to produce the film was iPP_MFI=2.5_, and multi-walled carbon nanotube-grade IGCNTs from Cheaptubes (Cambridgeport, VT, USA) with an outer diameter of 20–40 nm with a length of 10–30 μm and 90% purity.

### 2.2. Methods

The masterbatches were fabricated using melt extrusion ultrasound-assist methods designated as W-U, when no ultrasound assist was used; F-U and V-U, stand for fixed and variable frequency, respectively, when an ultrasound probe was attached at the exit of the die of the extruder [14,15].

Cast film of polypropylene was made by multilayer co-extrusion process at 260 °C. Three extruded layers were joined in a die to form a single structure. The film structure consisted of layers A/B/A (ABA), with a thickness 2.4/20/2.4 μm, respectively. The internal layer type B consisted of raw material of polypropylene (iPP_MFI=2.5_) and the external layers type A were fabricated with polypropylene homopolymer (iPP_MFI=2.5_, iPP_MFI=34_, iPP_MFI=1200_) and multi-wall carbon nanotubes (MWCNTs) at 0.01, 0.1, and 1 wt %, using the masterbatches described above with an appropriate dilution ratio. Thus, 3 different iPPs with 3 types of masterbatch fabrication and 3 different concentrations of MWCNTs rendered 27 ABA films. A reference film without MWCNTs was produced, were external layers were fabricated with homopolymer (iPP_MFI=2.5_).

### 2.3. Characterization Techniques

The electric and optical properties of the films were characterized. Additionally, morphology by optical and electron microscopy was obtained for selected samples to support the Discussion section.

### 2.4. Electrical Surface Resistivity

This property was measured on three stuck films of ABA reference film as well as on three stuck films containing MWCNTs with sample size of 9.5 × 9.5 cm^2^, using an Electrometer/High resistance meter from Keithley (Cleveland, OH, USA) model 6517B. A resistivity text fixture model 8009 from same company was attached to the Electrometer. A voltage of 1000 V was used for each measurement, and each sample was measured three times and the values averaged.

### 2.5. Electrostatic Charge

Electrostatic charge was evaluated with a portable electrostatic field meter, Simco-Ion model FMX-004 (Simco Ion, Industrial Group, Hatfiel, PA, USA), in the low range mode (0–3.00 kV). The three stuck films of each sample were used for electrical resistance. The samples were left to equilibrate for 2 h in a small open chamber protected from dust and draft at ambient conditions (23 °C, 40% RH, and an O_2_ partial pressure of ~135 mm Hg in Saltillo). Each sample was clamped to a grounded steel ring and a measurement of electrostatic charge was made. Then, each film was separated from the others and the electrostatic charge was measured immediately for each separated film.

### 2.6. Optical Properties

The color effect on the samples was recorded under the ASTM E 1347-06S standard using a CM-3600d Spectrophotometer Konica Minolta Sensing (Osaka, Japan). A white plate with color coordinates L = 98.91, a = −0.17, and b = −0.40 was used as the reference surface. Specular gloss of plastic films was evaluated under the ASTM D2457-97 standard using a Gloss meter Micro Tri Gloss BYK 20 60 85 (Schkopau, Germany). For the Haze analyses on the plastic films, ASTM test method D1003 was used with EEL 57D Sperical Hazemeter, Diffusion Systems Ltd. (Ealing, London, UK).

### 2.7. Optical Microscopy (OM)

OM was used to analyze the distribution of MWCNTs agglomerates in the PP/MWCNT ABA films with 0.1% and 1.0% of MWCNT, prepared using the masterbatch with MFI = 34 and the variable frequency ultrasound (V-U) method. The ABA films were observed in the transmittance mode in a digital microscope Model VHX-5000 from KEYENCE (Itasca, IL, USA).

### 2.8. Transmission Electron Microscopy (TEM)

TEM was used to analyze the MWCNT dispersion in the PP/MWCNT ABA film thin section (~90 nm) of the ABA film with 1.0% of MWCNT, which was prepared using the masterbatch with MFI = 34, and the variable frequency ultrasound (V-U) method, and cryo-microtoming. TEM micrographs were obtained using a TEM Titan 89-300 (FEI, Hillsboro, OR, USA) at an operating voltage of 300 keV.

### 2.9. Scanning Electron Microscopy (SEM)

SEM observations were used to determine the structure of MWCNT within the matrix of the polymer nanocomposites film. An ABA film with 1.0% of MWCNT, prepared using the masterbatch with MFI = 34 and the variable frequency ultrasound (V-U) method was cryo-fractured and coated with gold. The SEM observations were realized directly on the cryo-fractured surface of ABA film. SEM micrographs were obtained using a field emission scanning electron microscope, JSM-74101F JEOL VR (JEOL, Tokyo, Japan) with a secondary electron detector (SEI) using a voltage of 4.0 kV.

## 3. Results

Electrical and optical properties for ABA films were determined, and the results are shown as follows.

### 3.1. Electrical Properties

#### 3.1.1. Surface Resistivity

Electrical surface resistivity for ABA films are shown in Table 2. For the reference film, 0 wt % MWCNT, the surface resistivity measured was 2.1 × 10^17^ Ω/sq. In general, the surface resistivity of samples containing MWCNTs decreased with respect to the reference film, and fall within 10^12^ to 10^16^ Ω/sq, without a clear tendency with respect to the masterbatch fabrication method, with the MFI of iPP used to prepare the masterbatch or with the final MWCNT concentration on the A-layers of the films. In all cases, surface resistivity is within the range of insulator materials, above the range accepted for an antistatic film [11]. It must be kept in mind that the mechanism in this case is different from the well-known hydrophilic-mechanism, since hydrophobic MWCNTs are used to induce a decrease of electrical resistivity in the films.

#### 3.1.2. Electrostatic Charge

Electrostatic charge (kV) was measured in three films (F_1_, F_2_, and F_3_) that were stacked together. The initial charge of stacked films is shown in the Supporting File (Supplementary Materials), where it can be noticed that the initial charge is typically less than ±0.3 kV without any particular trend, and in an exceptional case, −0.79 kV. Once the films were unstuck, the electrostatic charge was measured for each one. Thus, three values are reported for a particular iPP, concentration of MWCNT in A-layers and fabrication method in Table 3, one for each film. The values obtained for two reference films are shown in the footnotes of the table.

On one hand, in the case of a commercial antistatic film, it can be noticed that the electrostatic charge is zero in two films, and −0.27 kV in one case where a minimum electrostatic charge, in this case close to 0 kV, is expected. On the other hand, films without any antistatic additives, fabricated only with hydrophobic polymers, show electrostatic charge values of 2.5, −3.5, and −0.76 kV. A large electrostatic charge is expected in this case and it can be fixed arbitrarily at 3.5 kV. Once the minimum (0 kV) and maximum (3.5 kV) values of electrostatic charge have been set, the results obtained with films containing MWCNT can be analyzed.

As in the case of surface resistivity shown above, there is no clear tendency or trend in the individual data. Therefore, a way to determine if electrostatic charge was affected in a significant manner is to find that the three values, one for each film, for a fixed MFI and concentration are below an arbitrary value of 1.17 kV (i.e., if one film shows a value above 1.17 kV it is considered that the sample was not significantly affected). This arbitrary value of 1.17 kV was chosen, taking into account that samples without antistatic additives show an electrostatic charge of 3.5 kV, and antistatic films 0 kV. Thus, a reduction of at least two third parts, 2.34 kV, can be considered significant (marked with grey background in Table 3). 

Keeping these considerations in mind, we note that the following observations can be made. All the films prepared with F-U masterbatch, regardless of the MFI of iPP and MWCNT concentration used, show at least one value above 1.0 kV. Therefore, a significant reduction of electrostatic charge was not found for films prepared using F-U masterbatches. A mixed behavior is observed in films prepared with W-U and V-U masterbatches. For example, films prepared with iPP_MFI=34_ show a significant reduction of electrostatic charge, while films prepared with iPP_MFI=2.5_ and iPP_MFI=1200_ do not. In the case of films prepared using V-U masterbatches, the situation is a bit complex, although 6 of 9 films show a significant reduction of electrostatic charge.

In conclusion, we note that films prepared using W-U masterbatch (iPP_MFI=34_) show significant reduction of electrostatic charge. The effect is more pronounced when MWCNT concentration is 0.01 wt %. Films prepared using V-U masterbatch show mixed behavior with respect to MWCNT concentration and iPP MFI used to prepare the masterbatch. Although, once more, the samples prepared with iPP_MFI=34_ show the highest effect. For films prepared with iPP_MFI=2.5_ only V-U masterbatches diluted to 0.01 wt % MWCNTs show a significant improvement. Finally, the use of F-U masterbatches does not improve the electrostatic charge behavior in a significant manner. Therefore, a combination of masterbatch preparation method, MFI of iPP, as well as MWCNT concentrations, play a key role towards the development of low electrostatic charge films.

### 3.2. Optical Properties

#### 3.2.1. Color Coordinates L*, a*, b*

According to the Commission International de l’Eclairage (CIE), the color of an object can be determined completely by using a three dimensional coordinate system (L*, a*, and b*) [16,17] where L* is the vertical coordinate, while a* and b* are the horizontal coordinates. Moreover, L* has values from 0 (black) to 100 (white), a* values ranging from −80 (green) to +80 (red), and b* ranging from −80 (blue) to +80 (yellow). The values of L*, a*, and b* for the reference film (0 wt %) were 98.78, −0.16, and −0.43, respectively, and the ones obtained for ABA films with MWCNTs are shown in Table 4.

From a general point of view, the addition of MWCNT to the A-layers shifts slightly the L* coordinate from white to black. The shift is slight for samples containing 0.01 and 0.1 wt % (L* values comparable to the reference film 98.78), i.e., a transparent film is obtained, while for samples containing 1 wt % of MWCNTs, the L* values ranged from 94 to 96, regardless of the fabrication method and of the MFI of iPP, i.e., with a greyish color. The values for coordinates a* and b* are close to zero. A shift from green to red is observed for coordinate a*, where the shifting is more pronounced when 1 wt % of MWCNTs is used. In a similar fashion, the use of 1 wt % MWCNTs generates a marked change in coordinate b*, shifting from blue to yellow. In both horizontal coordinates a* and b*, the use of minimum amounts of MWCNTs produces a slight variation. The shift in coordinates a* and b*, i.e., in samples with 0.01 and 0.1 wt % of MWCNTs does not change significantly. Therefore, the absence of color can be concluded from changes in coordinates a* and b*.

#### 3.2.2. Haze and Gloss

On the other hand, Haze and Gloss were also determined for the films with results presented in Table 5. Haze is defined as the percentage of light deviated from the incident beam 2.5°, i.e., the light that is diffused when passing through a transparent material, while Gloss is an optical property used to describe the visual appearance of an object and indicates how well a surface reflects light in a specular direction. For the reference film without MWCNT, Haze was 3.0.

In the case of samples containing 0.01 and 0.1 wt %, in general, a slight increase in Haze was observed up to 5.5, regardless of the masterbatch preparation method and MFI of iPP. A larger increase was detected for films containing 1 wt %, with values ranging from 4.7 to 8.7. Nonetheless, such increase is not marked in general terms. On the other hand, the Gloss for the reference film is 51.6, and is higher for samples containing 0.01 and 0.1 wt % with values ranging 66.2 to 89.1, where no preferred tendency regarding the MFI of PP and the masterbatch preparation was observed. Gloss values between 75 and 94.1 were obtained for films containing 1 wt %, where again, no preferred tendency was observed.

## 4. Discussion

**Figure 1:**
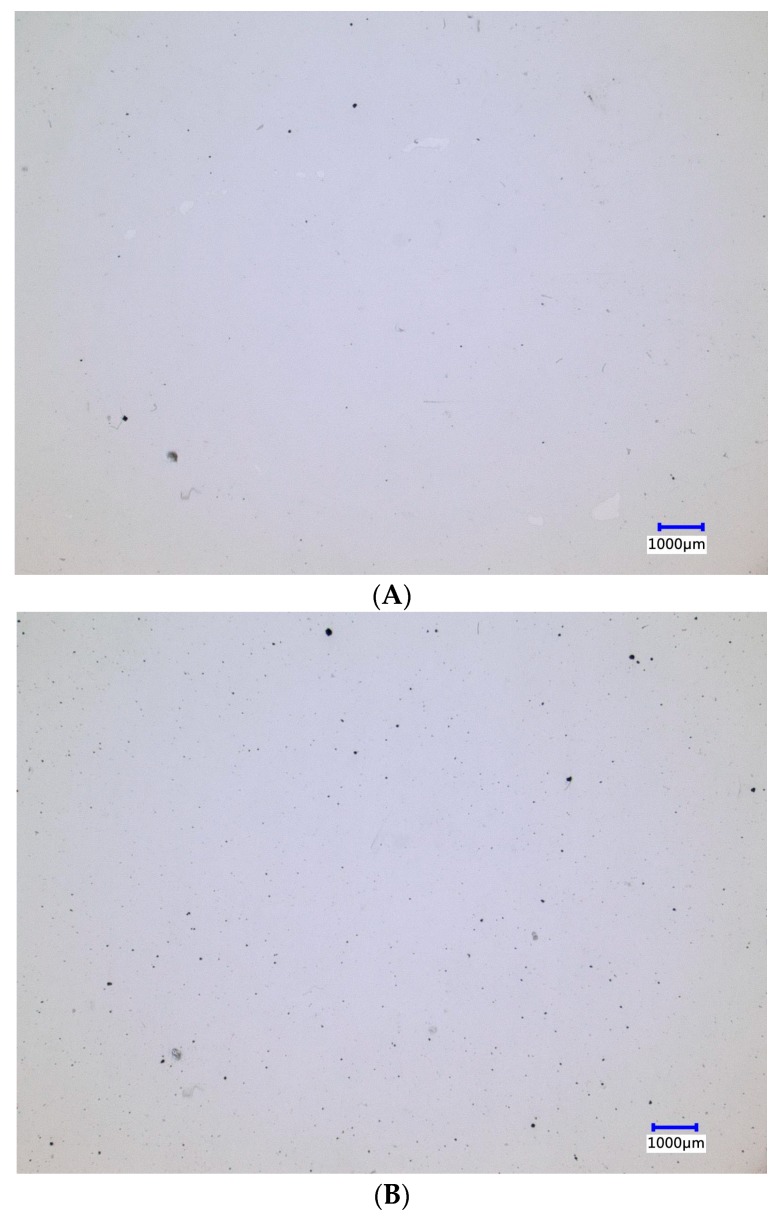
Optical micrographs for ABA films fabricated using masterbatch with MFI = 34 g/10 min. The masterbatches were fabricated using ultrasound-assist method V-U. (**A**) 0.1 wt % MWCNT; and (**B**) 1.0 wt % MWCNT in the A-layers.

**Figure 2:**
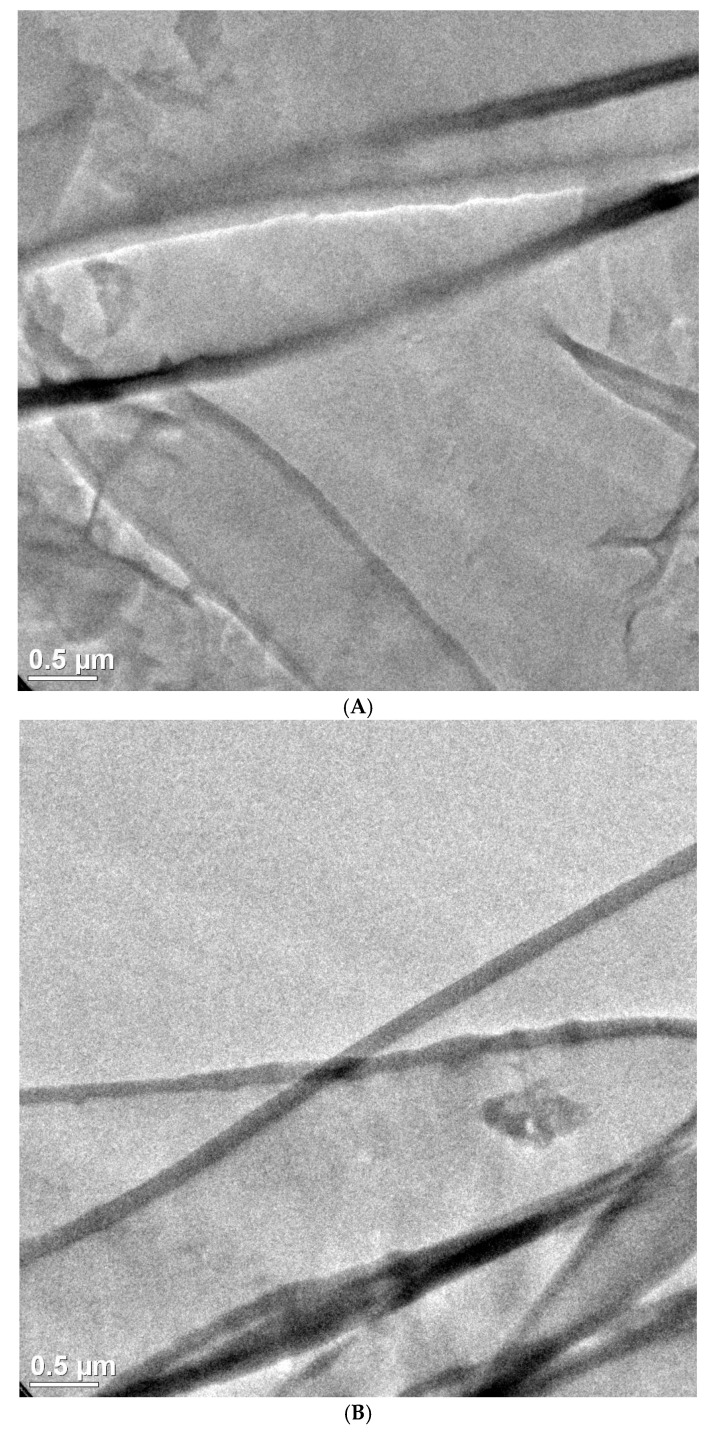
TEM micrographs for ABA film containing 1.0 wt % MWCNT in the A-layers fabricated using masterbatch with MFI = 34 g/10 min with ultrasound-assist method V-U. (**A**) MWCNTs near to each other, and (**B**) MWCNTs touching to each other.

According to the results, ABA films containing MWCNTs show electrical surface resistivity between 10^12^ and 10^16^ Ω/sq, regardless of the masterbatch fabrication method, MFI of iPP and MWCNT content, while the reference sample showed a surface resistivity of 10^17^ Ω/sq. Thus, all films prepared containing MWCNTs can be considered as insulating material, as is the case for the sample without MWCNTs. However, once the electrostatic charge was determined, only 9 films out of the 27 samples prepared showed a marked tendency to be less charged after being unstuck, where friction might be responsible for electrostatic charge of the films. Aiming to understand this behavior, we obtained optical and electron microscopy of selected samples showing low electrostatic charge. Optical micrographs of samples containing 0.1 and 1.0 wt % MWCNTs in the A-layers, fabricated using masterbatch with MFI = 34 g/10 min using ultrasound-assisted method V-U, are shown in Figure 1. An even distribution of agglomerates (black dots) can be found for both MWCNT concentrations selected. However, they are separated by a few microns from each other. In order to transport electrical charge, the MWCNTs must be touching one another (percolation mechanism) or at least closer than 2.1 nm (hopping mechanism) [18,19,20]. To investigate which is the predominant mechanism, we obtained TEM micrographs for samples containing 1.0 wt % MWCNTs in the A-layers, fabricated using masterbatch with MFI = 34 g/10 min using ultrasound-assist method V-U, as shown in Figure 2. It can be seen in two different fields of the samples that individual MWCNTs are closer to each other, and probably touching one to another. It is worth mentioning that finding individual MWCNTs was difficult, and large areas of the samples lack the presence of MWCNT. Therefore, the small region depicted in Figure 2 does not allow any conclusions to be drawn about the bulk transport mechanism. Figure 1 and Figure 2 show the presence of isolated nanotube clusters (Figure 1, black dots), as well as of isolated tubes, possibly forming a percolation network. Therefore, a percolation mechanism seems to be the preferred mechanism to transport electrical charge in the ABA-films. In this sense, it has been reported that the MWCNT agglomerates distribution, and dispersion of individual MWCNT,s play a key role in the reduction of electrical resistivity of BOPP films [13]. In this study, a significant reduction of electrical resistivity (in terms of insulator behavior) was not found, however, the electrostatic charge was diminished significantly by MWCNTs incorporation in to the external A-layer. This behavior can be associated with the contribution of individual MWCNTs from agglomerates during the preparation of ultrasound assisted masterbatches (V-U) [15]. The same effect is expected when iPP_MFI=34_ is used for the masterbatch fabrication, even in the absence of ultrasound waves. Thus, the presence of individual MWNTs can be a result of extrusion conditions and MFI favoring wetting and high shear forces.

**Figure 3:**
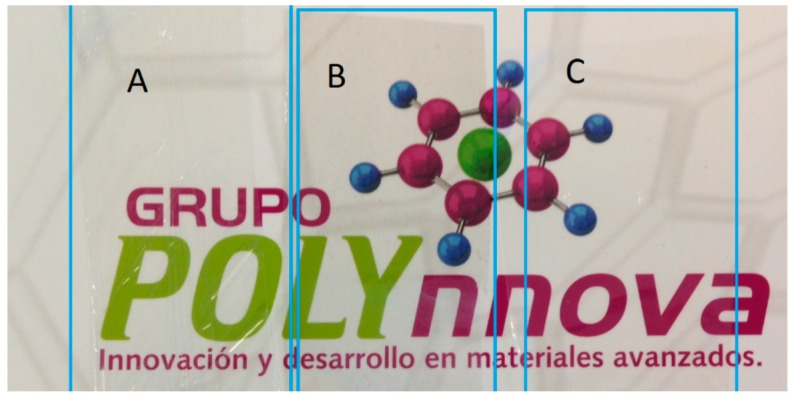
Photograph of ABA films: (**A**) reference film, 0 wt % MWCNT, (**B**) film with 1 wt % MWCNT (fabricated with masterbatch: iPP_MFI=2.5_, F-U), and (**C**) film with 0.01 wt % MWCNT (fabricated with masterbatch: iPP_MFI=2.5_, V-U).

On the other hand, the transparency of the ABA-films was high, with more than 98% whiteness (L*), and neglecting shifting on the coordinates a* and b*, for samples containing 0.01 and 0.1 wt % of MWCNTs. To have a better idea of the transparency of the films, photographs depicting different samples are shown in Figure 3. The samples without MWCNTs (Figure 3A) and with 0.01 wt % of MWCNTs (Figure 3C) show no significant difference, while the sample with 1.0 wt % shows a greyish color (Figure 3B).

**Figure 4:**
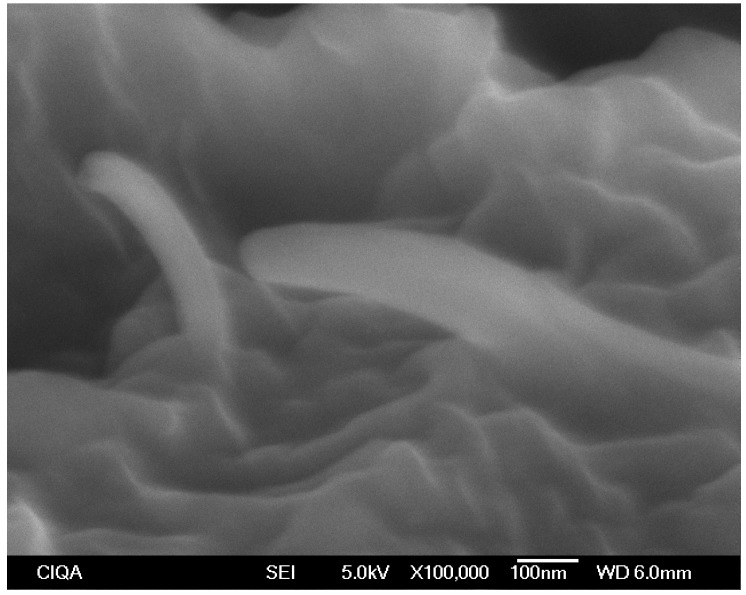
SEM micrographs for ABA film containing 1.0 wt % MWCNT in the A-layers fabricated using masterbatch with MFI = 34 g/10 min with ultrasound-assist method V-U.

In closing, we note an enhancement of brightness for the ABA-films containing MWCNTs. Brightness is a measurement of light reflectivity at a fixed angle. To our best knowledge, MWCNTs are powerful visible light absorbers, and we were not able to find any previous study reporting brightness enhancement or visible light reflection by MWCNTs embedded in a polymer matrix. However, it has been reported [21] that aligned carbon nanotubes can reflect visible light above 60°, a higher value than that the used in this study, where reflectivity is close to zero. In this case, we suggest that the iPP chains wrap MWCNTs, forming ordered regions of polymer chains on the surface of the nanotubes. These ordered regions, possibly crystallites, might be the ones responsible for the reflection of visible light, and therefore, of the brightness enhancement. The appearance of carbon nanotube wrapping by iPP molecules has been reported in the past [22], and it was observed also in this study, Figure 4, with an MWCNT with diameter of more than 100 nm. A number of such MWCNTs with diameters above 100 nm were observed. Nevertheless, MWCNTs diameter is ca. 50 nm as reported previously [14,15]. Thus, the increase of diameter was associated with iPP wrapping onto the MWCNT surface.

## 5. Conclusions

The incorporation of small amounts of MWCNT in A-layers of ABA polypropylene films lead to a decrease in the surface resistivity of the films of up to 5 orders of magnitude with respect to the pure iPP sample, still in the range of that for insulator materials. However, the static charge developed by the films after being separated changes significantly, depending on the melt flow index of iPP, and ultrasound assist method of fabrication of the masterbatch and content of MWCNT. Certain conditions, such as iPP MFI = 34 g/10 min in the absence of ultrasound assist and masterbatch fabricated using variable frequency ultrasound assist, at low concentrations for any given iPP MFI, seem to favor the presence of individual MWCNTs, which are responsible for the low electrostatic charge. Besides, wrapping of individual MWCNTs might be responsible for the bright enhancement of the films. Finally, small amounts of MWCNT and its dispersion within the polymer matrix are essential to obtain transparent films.

## Figures and Tables

**Table 1:** iPP characteristics.

Code	MFI (g/10 min)	Provider	Grade
iPP_MFI=2.5_	2.5	Exxon (Houston, TX, USA)	PP4712
iPP_MFI=34_	34	Indelpro (Altamira, Mexico)	PL835N
iPP_MFI=1200_	1200	Indelpro (Altamira, Mexico)	Profax PL 505

MFI: Melt flow index.

**Table 2:** Surface resistivity (Ω/sq) for ABA films fabricated using masterbatches with MFI = 2.5, 34, and 1200 g/10 min. The masterbatches were fabricated using ultrasound-assist methods: W-U, F-U, and V-U.

wt % MWCNT in A-Layers	iPP_MFI=2.5_	iPP_MFI=34_	iPP_MFI=1200_
W-U			
0.01	4.2 × 10^14^	8.6 × 10^14^	2.5 × 10^15^
0.10	1.1 × 10^15^	1.0 × 10^15^	4.0 × 10^16^
1.00	8.3 × 10^13^	2.6 × 10^12^	3.6 × 10^16^
F-U			
0.01	3.0 × 10^15^	2.8 × 10^14^	1.6 × 10^13^
0.10	1.2 × 10^16^	6.0 × 10^15^	8.0 × 10^13^
1.00	1.2 × 10^13^	1.2 × 10^14^	1.3 × 10^16^
V-U			
0.01	9.4 × 10^14^	2.2 × 10^15^	4.0 × 10^14^
0.10	5.1 × 10^15^	2.0 × 10^14^	1.4 × 10^14^
1.00	6.0 × 10^14^	5.3 × 10^12^	1.8 × 10^14^

Commercial antistatic film, surface resistivity = 1.0 × 10^11^ Ω/sq; Reference film, 0 wt % MWCNT, surface resistivity = 2.1 × 10^17^ Ω/sq.

**Table 3:** Electrostatic charge (kV) for unstuck ABA films fabricated using masterbatches with MFI = 2.5, 34 and 1200 g/10 min. The masterbatches were fabricated using ultrasound-assist methods: W-U, F-U and V-U.

wt % MWCNT in A-Layers	iPP_MFI=2.5_			iPP_MFI=34_			iPP_MFI=1200_		
	*F*_1_	*F*_2_	*F*_3_	*F*_1_	*F*_2_	*F*_3_	*F*_1_	*F*_2_	*F*_3_
W-U									
0.01	0.87	−1.60	0.73	−0.16	−0.46	0.27	−1.19	−1.24	0.48
0.10	0.94	−1.61	0.73	0.62	−0.89	−0.01	2.40	−0.27	−1.80
1.00	0.50	−1.40	−2.50	0.53	−0.34	0.01	1.90	−1.80	0.97
F-U									
0.01	0.22	0.07	1.60	0.45	0.77	−1.20	−2.40	0.92	−0.69
0.10	−0.92	−1.50	0.21	1.38	−2.10	2.60	−0.08	−0.51	−3.60
1.00	0.92	−4.60	1.22	−0.77	1.26	−0.35	−2.30	0.41	1.25
V-U									
0.01	0.03	−0.87	0.19	0.37	−0.34	1.17	−0.19	−0.79	0.38
0.10	−4.20	−3.60	0.67	−0.50	−0.06	−0.37	−0.78	−0.32	0.40
1.00	0.50	−3.70	1.28	−0.20	−0.16	0.07	−3.70	3.80	−1.50

Commercial antistatic film, electrostatic charge (kV) for each unstuck film *F*_1_ = 0.00, *F*_2_ = −0.27, and *F*_3_ = 0.00; Reference film, 0 wt % MWCNT, electrostatic charge (kV) for each unstuck film *F*_1_ = 2.50, *F*_2_ = −3.50, and *F*_3_ = 0.76.

**Table 4:** Color coordinates L*, a*, and b* for ABA films fabricated using masterbatches with MFI = 2.5, 34 and 1200 g/10 min. The masterbatches were fabricated using ultrasound-assist methods: W-U, F-U and V-U.

wt % MWCNT in A-Layers	iPP_MFI=2.5_			iPP_MFI=34_			iPP_MFI=1200_		
	L*	a*	b*	L*	a*	b*	L*	a*	b*
W-U									
0.01	98.68	−0.16	−0.38	98.64	−0.16	−0.38	98.64	−0.16	−0.39
0.10	98.26	−0.15	−0.30	98.22	−0.14	−0.29	98.18	−0.15	−0.27
1.00	94.71	−0.07	0.25	94.00	−0.05	0.41	94.14	−0.05	0.37
F-U									
0.01	98.68	−0.17	−0.38	98.72	−0.17	−0.40	98.68	−0.16	−0.41
0.10	98.34	−0.16	−0.32	98.13	−0.14	−0.27	98.28	−0.14	−0.30
1.00	94.34	−0.04	0.29	93.89	−0.04	0.42	94.45	−0.05	0.34
V-U									
0.01	98.62	−0.16	−0.38	98.64	−0.15	−0.40	98.69	−0.15	−0.41
0.10	98.22	−0.14	−0.32	98.10	−0.13	−0.28	98.30	−0.14	−0.33
1.00	96.39	−0.03	0.01	95.19	−0.05	0.21	95.56	−0.06	0.17

Reference film, 0 wt % MWCNT, L* = 98.78, a* = −0.16, b* = −0.43.

**Table 5:** Haze and brightness for ABA films fabricated using masterbatches with MFI = 2.5, 34 and 1200 g/10 min. The masterbatches were fabricated using ultrasound-assist methods: W-U, F-U and V-U.

wt % MWCNT in A-Layers	iPP_MFI=2.5_		iPP_MFI=34_		iPP_MFI=1200_	
	Haze (%)	Gloss	Haze	Gloss	Haze	Gloss
W-U						
0.01	2.9	66.2	3.6	70.2	3.9	76.3
0.10	3.9	89.1	3.9	80.9	4.7	81.6
1.00	5.6	75.7	7.0	78.4	8.3	80.3
F-U						
0.01	4.9	78.4	3.9	76.5	3.8	77.5
0.10	4.9	77.6	4.8	73.8	5.1	73.7
1.00	8.6	94.1	8.7	83.1	7.6	80.8
V-U						
0.01	5.5	77.5	4.4	78.6	4.9	75.8
0.10	4.8	82.8	4.8	75.4	4.7	74.7
1.00	4.7	78.1	6.5	79.2	5.6	84.3

Reference film, 0 wt % MWCNT. Haze = 3.0. Gloss = 51.6.

## Supplementary Materials

The following are available online at www.mdpi.com/2073-4360/10/1/55/s1, Table S1: Electrostatic charge (kV) prior to unstuck for 3 stacked ABA films fabricated using masterbatches with MFI = 2.5, 34 and 1200 g/10 min. The masterbatches were fabricated using ultrasound-assist methods: W-U, F-U and V-U.

## References

[1] Swogger K.W., Poon B., Stephens C.H., Ansems P., Chum S., Hiltner A., Baer E. Material classification and applications of new propylene-ethylene copolymers. Proceedings of the ANTEC 2003: Annual Technical Conference.

[2] Chow W.S., Tham W.L. (2009). Effects of antistatic agent on the mechanical, morphological and antistatic properties of polypropylene/organo-montmorillonite nanocomposites. Express Polym. Lett..

[3] Li C., Liang T., Lu W., Tang C., Hu X., Cao M., Liang J. (2004). Improving the antistatic ability of polypropylene fibers by inner antistatic agent filled with carbon nanotubes. Compos. Sci. Technol..

[4] Valdez-Garza J., Avila-Orta C., Cruz-Delgado V., Gonzalez-Morones P., Hurtado-Lopez G., Waldo-Mendoza M., Quinones-Jurado Z., Perez-Medina J. (2017). Antistatic films based on polymer nanocomposites. Boletín del Grupo Español del Carbón.

[5] Iijima S. (1991). Helical microtubules of graphitic carbon. Nature.

[6] Dürkop T., Getty S.A., Cobas E., Fuhrer M.S. (2004). Extraordinary mobility in semiconducting carbon nanotubes. Nano Lett..

[7] Novoselov K.S., Geim A.K., Morozov S.V., Jiang D., Zhang Y., Dubonos S.V., Grigorieva I.V., Firsov A.A. (2004). Electric field effect in atomically thin carbon films. Science.

[8] Seo M.K., Park S.J. (2004). Electrical resistivity and rheological behaviors of carbon nanotubes-filled polypropylene composites. Chem. Phys. Lett..

[9] McLachlan D.S., Chiteme C., Park C., Wise K.E., Lowther S.E., Lillehei P.T., Siochi E.J., Harrison J.S. (2005). Ac and dc percolative conductivity of single wall carbon nanotube polymer composites. J. Polym. Sci. B.

[10] Bauhofer W., Kovacs J.Z. (2009). A review and analysis of electrical percolation in carbon nanotube polymer composites. Compos. Sci. Technol..

[11] Shen J.B., Champagne M.F., Yang Z., Yu Q., Gendron R., Guo S.Y. (2012). The development of a conductive carbon nanotube (CNT) network in CNT/polypropylene composite films during biaxial stretching. Compos. A Appl. Sci. Manuf..

[12] You F., Li X., Zhang L., Wang D., Shi C., Dang Z. (2017). Polypropylene/poly(methyl methacrylate)/graphene composites with high electrical resistivity anisotropy via sequential biaxial stretching. RSC Adv..

[13] Shen J.B., Champagne M.F., Gendron R., Guo S.Y. (2012). The development of conductive carbon nanotube network in polypropylene-based composites during simultaneous biaxial stretching. Eur. Polym. J..

[14] Ávila-Orta C.A., Quiñones-Jurado Z.V., Waldo-Mendoza M.A., Rivera-Paz E.A., Cruz-Delgado V.C.J., Mata-Padilla J.M., González-Morones P., Ziolo R.F. (2015). Ultrasound-assist extrusion methods for the fabrication of polymer nanocomposites based on polypropylene/multi-wall carbon nanotubes. Materials.

[15] Pérez-Medina J.C., Waldo-Mendoza M.A., Cruz-Delgado V.J., Quiñones-Jurado Z.V., González-Morones P., Ziolo R.F., Martínez-Colunga J.G., Soriano-Corral F., Avila-Orta C.A. (2016). Metamaterial behavior of polymer nanocomposites based on polypropylene/multi-walled carbon nanotubes fabricated by means of ultrasound-assisted extrusion. Materials.

[16] Mc Laren K. (1986). The Colour Science of Dyes and Pigments.

[17] Judd D.B., Wyszecki G. (1975). Colour in Bussines, Science and Industry.

[18] Gong S., Zhu Z.H., Li Z. (2017). Electron tunnelling and hopping effects on the temperature coefficient of resistance of carbon nanotube/polymer nanocomposites. Phys. Chem. Chem. Phys..

[19] Zhou Y., Azumi R. (2016). Carbon nanotube based transparent conductive films: Progress, challenges, and perspectives. Sci. Technol. Adv. Mater..

[20] Ventura I.A., Zhou J., Lubineau G. (2015). Investigating the inter-tube conduction mechanism in polycarbonate nanocomposites prepared with conductive polymer-coated carbon nanotubes. Nanoscale Res. Lett..

[21] Wąsik M., Judek J., Zdrojek M. (2013). Polarization-dependent optical reflection from vertically aligned multiwalled carbon nanotube arrays. Carbon.

[22] Valentini L., Biagiotti J., Kenny J.M., Santucci S. (2003). Morphological characterization of single-walled carbon nanotubes-pp composites. Compos. Sci. Technol..

