# Supplementary material for: Transparent Low Electrostatic Charge Films Based on Carbon Nanotubes and Polypropylene. Homopolymer Cast Films
Source: Polymers (Basel). 2018 Jan 9;10(1):55. doi: 10.3390/polym10010055 (PMC6415039; doi:10.3390/polym10010055)
Supplement: Supplementary file 1 [file polymers-10-00055-s001.zip › Antistatic Cast Film 20171125 SI.docx]

Transparent low electrostatic charge films based on carbon nanotubes and polypropylene. Homopolymer cast films

Zoe V Quiñones-Jurado^1^, Miguel A Waldo-Mendoza^1^, José M Mata-Padilla^2^, Pablo González-Morones^3^, Juan Guillermo Martínez-Colunga^3^, Florentino Soriano-Corral^3^, Víctor J Cruz-Delgado^3,*^, Ronald F Ziolo^3^ and Carlos A Avila-Orta^3,*^

**Table S1.** Electrostatic charge (kV) prior to unstuck for 3 stacked ABA films fabricated using masterbatches with MFI = 2.5, 34 and 1200 g/10 min. The masterbatches were fabricated using ultrasound-assisted methods: W-U, F-U and V-U.

| % wt/wt MWCNT in A-layers | iPP_MFI=2.5_ | iPP_MFI=34_ | iPP_MFI=1200_ |
| --- | --- | --- | --- |
| W-U | | | |
| 0.01 | -0.05 | -0.07 | -0.21 |
| 0.10 | -0.07 | -0.02 | -0.14 |
| 1.00 | -0.08 | 0.15 | -0.10 |
| F-U | | | |
| 0.01 | 0.09 | 0.13 | 0.02 |
| 0.10 | -0.19 | -0.15 | -0.02 |
| 1.00 | -0.01 | -0.11 | -0.11 |
| V-U | | | |
| 0.01 | -0.79 | -0.31 | -0.37 |
| 0.10 | -0.06 | -0.30 | -0.14 |
| 1.00 | 0.21 | -0.07 | 0.09 |
| PT | | | |
| 0.01 | -0.10 | -0.23 | -0.38 |
| 0.10 | -0.19 | -0.11 | -0.25 |
| 1.00 | -0.33 | -0.26 | -0.06 |

Commercial antistatic film, electrostatic charge = -0.05 kV

Reference film, 0 wt% MWCNT, electrostatic charge = -0.05 kV
